# Depression and Age-Related Changes in Body Composition, Cardiovascular Function, Grip Strength, and Lung Function

**DOI:** 10.1093/geroni/igab046.1709

**Published:** 2021-12-17

**Authors:** Julian Mutz, Cathryn Lewis

**Affiliations:** King's College London, London, England, United Kingdom

## Abstract

Individuals with mental disorders, on average, die prematurely, have higher levels of physical comorbidities and may experience accelerated ageing. In individuals with lifetime depression and healthy controls, we examined associations between age and multiple physiological measures. The UK Biobank study recruited >500,000 participants, aged 37-73, between 2006–2010. Generalised additive models were used to examine associations between age and grip strength, cardiovascular function, body composition, lung function and bone mineral density. Analyses were conducted separately in males and females with depression compared to healthy controls. Analytical samples included up to 342,393 adults (mean age = 55.87 years; 52.61% females). We found statistically significant differences between individuals with depression and healthy controls for most physiological measures, with standardised mean differences between -0.145 and 0.156. There was some evidence that age-related changes in body composition, cardiovascular function, lung function and heel bone mineral density followed different trajectories in individuals with depression. These differences did not uniformly narrow or widen with age. For example, BMI in female cases was 1.1 kg/m2 higher at age 40 and this difference narrowed to 0.4 kg/m2 at age 70. In males, systolic blood pressure was 1 mmHg lower in cases at age 45 and this difference widened to 2.5 mmHg at age 65. Individuals with depression differed from healthy controls across a broad range of physiological measures. Differences in ageing trajectories differed by sex and were not uniform across physiological measures, with evidence of both age-related narrowing and widening of case-control differences.

